# Metagenomics-Based Analysis of the Age-Related Cumulative Effect of Antibiotic Resistance Genes in Gut Microbiota

**DOI:** 10.3390/antibiotics10081006

**Published:** 2021-08-20

**Authors:** Lei Wu, Xinqiang Xie, Ying Li, Tingting Liang, Haojie Zhong, Jun Ma, Lingshuang Yang, Juan Yang, Longyan Li, Yu Xi, Haixin Li, Jumei Zhang, Xuefeng Chen, Yu Ding, Qingping Wu

**Affiliations:** 1 School of Food and Biological Engineering, Shaanxi University of Science and Technology, Xi’an 710021, China; wuleigdim@163.com (L.W.); gdim_liangtt@outlook.com (T.L.); majun@sust.edu.cn (J.M.); yj18185238563@163.com (J.Y.); chenxf@sust.edu.cn (X.C.); 2 Guangdong Provincial Key Laboratory of Microbial Safety and Health, State Key Laboratory of Applied Microbiology Southern China, Institute of Microbiology, Guangdong Academy of Sciences, Guangzhou 510070, China; woshixinqiang@126.com (X.X.); liying@gdim.cn (Y.L.); yangls8272@163.com (L.Y.); 18868006204@163.com (L.L.); xiyu_0604@163.com (Y.X.); 201920146453@mail.scut.edu.cn (H.L.); zhangjm926@126.com (J.Z.); 3 The First Affiliated Hospital, School of Clinical Medicine of Guangdong Pharmaceutical University, Guangzhou 510080, China; jaxzhong@126.com; 4 Department of Food Science and Technology, Institute of Food Safety and Nutrition, Jinan University, Guangzhou 510632, China

**Keywords:** metagenomics, gut microbiota, antibiotic resistance genes, longevity people, cumulative effect

## Abstract

Antibiotic resistance in bacteria has become a major global health problem. One of the main reservoirs of antibiotic resistance genes is the human gut microbiota. To characterise these genes, a metagenomic approach was used. In this study, a comprehensive antibiotic resistome catalog was established using fecal samples from 246 healthy individuals from world’s longevity township in Jiaoling, China. In total, 606 antibiotic resistance genes were detected. Our results indicated that antibiotic resistance genes in the human gut microbiota accumulate and become more complex with age as older groups harbour the highest abundance of these genes. Tetracycline resistance gene type *tetQ* was the most abundant group of antibiotic resistance genes in gut microbiota, and the main carrier of antibiotic resistance genes was *Bacteroides*. Antibiotic efflux, inactivation, and target alteration were found to be the dominant antimicrobial resistance mechanisms. This research may help to establish a comprehensive antibiotic resistance catalog that includes extremely long-lived healthy people such as centenarians, and may provide potential recommendations for controlling the use of antibiotics.

## 1. Introduction

Since the discovery of penicillin in 1929 [1], antibiotic resistance in bacteria has become an increasing threat to human health and a global health problem [2]. The emergence of antibiotic-resistant pathogens such as the New Delhi metallo-β-lactamase superbug [3], the carbapenem-resistant *Klebsiella pneumoniae* [4], multidrug-resistant *Mycobacterium tuberculosis* [5], and methicillin-resistant *Staphylococcus aureus* (MRSA) [6] has presented a major impact on human health. It is generally believed that the emergence and rapid spread of antibiotic resistance in the microbiota can be mainly attributed to the abuse of antibiotics by humans [7].

Antibiotics can have several effects on the human gut microbiota, which is a complex and dynamic equilibrium ecosystem [8]. When exposed to antibiotics, the microbiota not only responds through its own resistance mechanisms, but also optimises and spreads antibiotic resistance genes (ARG) through transformation, transfer, and recombination, and forms a colony with antibiotic resistance phenotype [9]. This antibiotic-induced disruption of microbiota may cause various diseases such as diabetes [10,11,12], neurological disorders [13], obesity [14,15], inflammation [16], and infections [17]. The human gut microbiota is considered to be the reservoir of ARG [18], which can quickly and easily exchange these genes to spread drug resistance [19]. This presents a high risk of increased antibiotic resistance in human pathogens [20], which has become a serious global public health problem as it renders previously reliable antibiotics ineffective.

Different methods have been used to characterise ARG in human gut microbiota, including isolation of antibiotic-resistant strains [21], polymerase chain reaction (PCR) based on specific primers, high-throughput quantitative PCR (qPCR) [22], microarray analysis [23] and metagenomics [24,25,26,27]. At present, metagenomic analysis based on high-throughput sequencing is commonly used in studying ARG due to its high efficiency and excellent characteristics [28,29]. Just as the human microbiome can constitute a mobile ARG reservoir, pathogens can use these genes to obtain antibiotic resistance through gene transfer [30]. Reference databases such as the Antibiotic Resistance Genes Database (ARDB) [31] and the Comprehensive Antibiotic Resistance Database (CARD) [32] have been developed to help researchers investigate ARG, such as those in the intestinal flora of Chinese, Danish, and Spanish populations. The difference in antibiotic resistance can be attributed to the different use and selection pressure of antibiotics in different countries [33]. However, the characteristics of ARG over time has not been clearly characterized. In particular, the relationship between the ARG in gut microbiota of longevity people and age remains unknown.

To address this problem, we analysed the ARG in the gut microbiota of 246 individuals from the world’s longevity township Jiaoling in China. Our results indicated that ARG in the human gut microbiota accumulate and become more complex with age given that older groups harbour the highest abundance of these genes. *Bacteroides* was found to be the main carrier of ARG in the human gut microbiota, of which tetracycline antibiotics resistance gene type *tetQ* was the most abundant group. Antibiotic efflux, inactivation, and target alteration were the dominant resistance mechanisms. This study will help establish a comprehensive list of antibiotic resistance in healthy and long-lived people, and provide a useful reference for the management of human ARG.

## 2. Materials and Methods

### 2.1. Study Cohort and Sample Collection

The overall research objective of the Jiaoling (world’s longevity township, Jiaoling County, Meizhou City, Guangdong Province, China) cohort is to study how ARG in the gut microbiota changes with age and how this affects health. The study was approved by the Ethics Committee of The First Affiliated Hospital/School of Clinical Medicine of Guangdong Pharmaceutical University.

From this cohort, 246 participants were randomly recruited from eight towns in June 2019. Participants must meet the following conditions: (1) born in Jiaoling; (2) have lived in Jiaoling for five consecutive years since the time of sampling; and (3) all age groups. All selected participants signed an informed consent form before the physical examination and biological material collection. To proceed to the metagenomic study, additional criteria were employed: (1) has fecal samples; (2) did not undergo antibiotic treatment within one month before the biological material was collected; and (3) no severe disease (diabetes, cancer, etc.). All participants met these requirements. Fecal samples were freshly collected from each subject and immediately frozen at −20 °C, transported to the laboratory on an ice pack, and stored at −80 °C until analysis.

### 2.2. DNA Extraction

Genomic DNA was extracted according to the manufacturer’s instructions (Magen, Stool DNA Kit, Guangzhou Magen Biotechnology Co., Ltd., Guangzhou, China) with some modification. Briefly, 1 mL of STL buffer was added to a 0.25–1 g sample and vortexed with glass beads for 15–20 min. It was then centrifuged at 12,000× *g* for 20 min, and the supernatant was transferred to a new 2-mL tube. In addition, 160 µL PS buffer and 160 µL absorbent solution were added. After centrifugation at 12,000× *g* for 10 min, the supernatant was transferred to a new 2-mL tube, and 650 μL GDP buffer was added. The column was used to filter the product, and then the product followed by DNA elution with 200 μL of sterile water.

### 2.3. Metagenomic Sequencing and Data Quality Control

The samples were sequenced using an Illumina HiSeq PE150 platform (Beijing Novogene Technology Co., Ltd., Beijing, China). The following standards were used for quality control: (1) reads were removed that contained low-quality bases (quality value ≤ 38) exceeding a certain percentage (default is 40 bp); (2) N bases were removed to reach a certain proportion of reads (default is 10 bp); (3) reads were removed whose overlap with the adapter exceeded a certain threshold (default is 15 bp); (4) if the sample had human contamination, it was compared with the human sequence to filter out the possible source of the human reads [34,35,36]. Bowtie2 software was used by default.

### 2.4. Metagenomic Assembly

After preprocessing, clean data were obtained and SOAPdenovo software [9] was used for assembly analysis. For the single sample assembly, parameters were: -d 1, -M 3, -R, -u, -F [37,38,39]. The assembled Scaffolds were broken from the N junction to obtain sequence fragments without N, called Scaftigs (i.e., continuous sequences within scaffolds) [40]. Bowtie2 software was used to compare the Clean Data after quality control of each sample to the assembled Scaftigs of each sample to acquire unused PE reads. The unused reads of each sample were then put together, and K-mer = 55 was selected for mixed assembly [41]. For Scaftigs generated by single sample and mixed assembly, fragments below 500 bp were filtered out [42,43,44].

### 2.5. Gene Catalog Construction

Starting from each sample- and mixed-assembled Scaftigs, MetaGeneMark was used for ORF (Open Reading Frame) prediction [45,46,47]. For the ORF prediction results of each sample and hybrid assembly, CD-HIT software was used for de-redundancy to obtain a non-redundant initial gene catalogue [48,49]. The clean data of each sample were compared to the initial gene catalogue using Bowtie2, and the number of reads on the gene comparison was calculated in each sample. The genes that support the number of reads ≤ 2 in each sample were filtered out, and the gene catalogue (unigenes) was obtained for subsequent analysis.

### 2.6. Species Annotation

(1) DIAMOND [50] software (V0.9.9, https://github.com/bbuchfink/diamond/) (accessed on 17 November 2020) was used to blast the unigenes to the sequences of Bacteria which are all extracted from the NR database (Version: 2018-01-02, https://www.ncbi.nlm.nih.gov/) (accessed on 17 November 2020) of NCBI. (2) The LCA algorithm which was applied to system classification of MEGAN [51] software was taken to make sure the species annotation information of sequences. (3) The table containing the number of genes and the abundance information of each sample in each taxonomy hierarchy (kingdom, phylum, class, order, family, genus, species) were obtained based on the LCA annotation result and the gene abundance table.

### 2.7. Analysis of Antibiotic Resistance Genes

The core component of the CARD database is Antibiotic Resistance Ontology (ARO), which integrates information such as sequence, antibiotic resistance, mechanism of action, and associations between AROs, and provides online interfaces between ARO and PDB, NCBI and other databases [52]. The basic steps of resistance gene annotation were as follows: (1) Resistance Gene Identifier software (RGI has built-in blastp, and uses the bitscore value to compare the results to score [53]) was used to compare unigenes; (2) Starting from the abundance of ARO, an abundance bar graph display, an abundance cluster heat map display, an abundance distribution circle graph display, and resistance gene species attribution analysis (annotate to unigenes of ARO) were performed. For part of the ARO with a long name, the first three words and an underscore were used to display the abbreviation. Finally, there were a total of 5,364,988 ORFs after the original de-redundancy, 3096 genes were compared to the CARD database, and a total of 606 types of ARO were included.

### 2.8. Statistical Analysis

Statistical analysis was implemented using the R platform and SPSS 16.0 software (SPSS Inc., Chicago, IL, USA). The “ggplot2” package and GraphPad Prism 8 software (GraphPad Software Inc., San Diego, CA, USA) were used to visualize. SOAPdenovo software [9] was used for assembly analysis. DIAMOND software [50] was used to blast. MEGAN software [51] was used to make sure the species annotation information of sequences. The Wilcoxon rank-sum test was used to evaluate the significance of differences in six groups. * for *p* < 0.05; ** for *p* < 0.01; *** for *p* < 0.001.

## 3. Results

### 3.1. Abundance of Antibiotic Resistance Genes Is an Age-Related Cumulative Effect

We established 5.36 million human gut microbiota genes from the sequencing data of 246 individuals. The subjects were divided into six age groups, Y20 group (0–20 years old), Y40 group (21–40 years old), Y60 group (41–60 years old), Y80 group (61–80 years old), Y100 group (81–100 years old), Y120 group (100–120 years old). Of these, 3096 unique ARG were found after the original de-redundancy. These genes account for 0.057% of the total genes of the human gut microbiota. This is higher than the 0.026% reported in the previous literature in 2013 [33]. Similarly, compared with other natural environments (including soil, ocean, lake, etc.), antibiotic resistance genes are obviously very abundant in the human gut microbiota [33].

**Figure 1:**
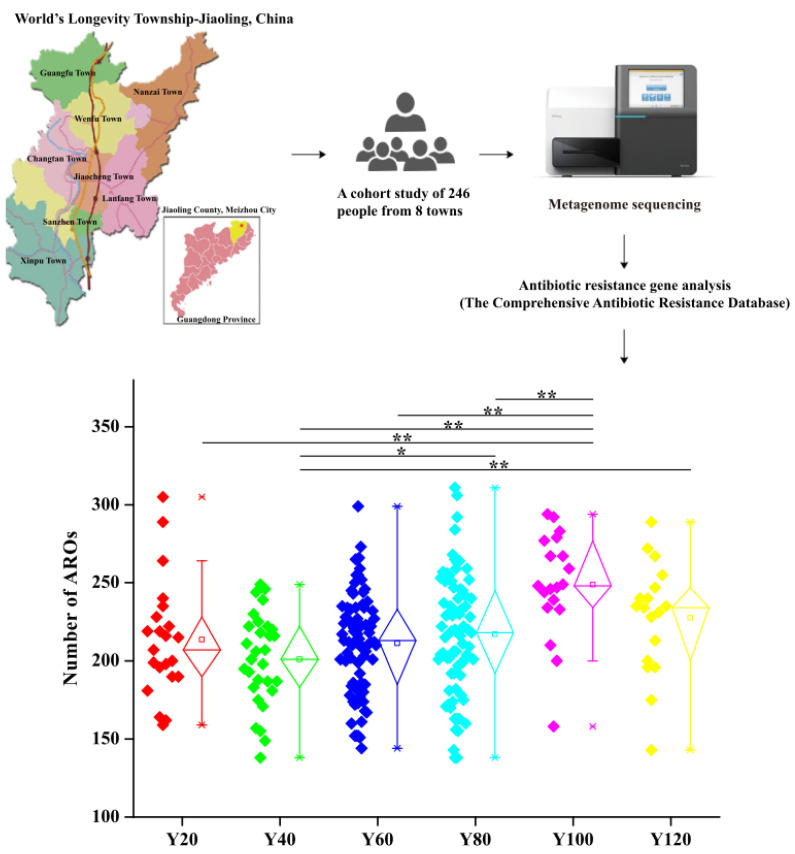
Comparison of the abundance of ARG in each group. The levels of significance for the Wilcoxon rank-sum test are: * for *p* < 0.05; ** for *p* < 0.01.

To compare the ARG abundances of different age groups, we calculated the number of ARG in each group based on sequencing coverage. The number of resistance genes in the older group (Y100) was significantly higher than that in the younger groups (Y20, Y40 and Y60; Figure 1). There is no difference between Y20–Y60 and that, from Y100, Y120, the number decreased (Figure 1). Our results indicate that ARG in the human gut microbiota accumulate and become more complex with age, with older groups harbouring the highest abundance of these genes.

### 3.2. Representative Resistance Gene Types in Different Age Groups

**Figure 2:**
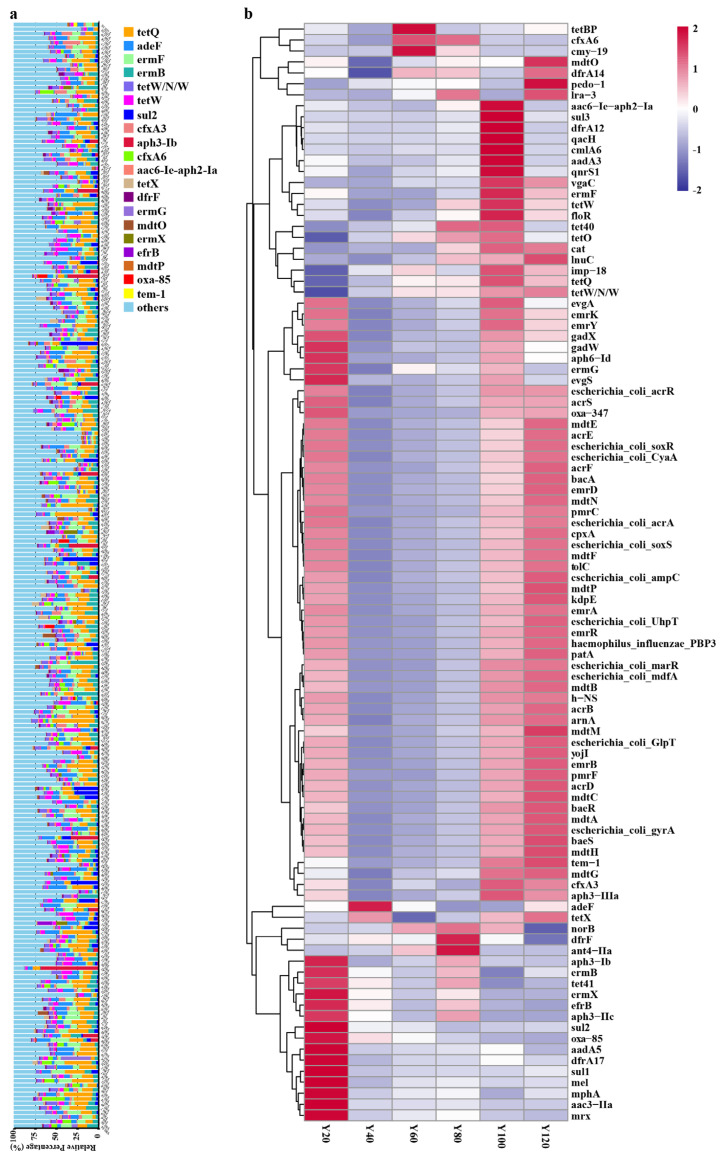
Comparative analysis of ARG in different age groups. (**a**) comparison of the top 20 most abundant ARG types in differently-aged individuals; (**b**) comparison of the top 100 most abundant ARG types in the different age groups.

The top twenty most abundant antibiotic resistance gene types varied among the different age groups (Figure S1) with the exception of *tetQ*, which was the most abundant type in all groups (Figure 2a). Previous literature has shown that the prevalence of the *tetQ* gene in *Bacteroides* isolates has nearly tripled [54]. The mechanism of tetracycline resistance gene type *tetQ* is antibiotic target protection, and *tetQ* belongs to the gene family of tetracycline-resistant ribosomal protection proteins (Table 1). The second most abundant gene type was the fluoroquinolone and tetracycline (FT) resistance gene type *adeF* (Figure 2a). It operates through antibiotic efflux and belong to the gene family of resistance–nodulation–cell division (RND) efflux pumps (Table 1). *ermF* and *ermB* were the third and fourth most abundant the gene types, respectively (Figure 2a). They are macrolide–lincosamide–streptogramin B (MLS) resistance genes that alter the antibiotic target and belong to gene family of erm 23S ribosomal RNA methyltransferases (Table 1). We found that the mechanisms of ARG in *Proteobacteria*, *Firmicutes*, and *Bacteroidetes* are mainly antibiotic efflux, inactivation, and target alteration. Antibiotic efflux is mainly present in *Proteobacteria*, while antibiotic inactivation and target alteration mainly occur in *Firmicutes* (Figure S2).

In the different age groups, different representative resistance gene types were observed (Figure 2b). *aph3−Ib,* *ermB, tet41*, *ermX*, *efrB*, *aph3−IIc*, *sul2*, *oxa−85*, *aadA5*, *dfrA17*, *sul1*, *mel*, *mphA*, *aac3−IIa*, and *Mrx* resistance gene types have the highest abundance in the Y20 group. Meanwhile, *adeF, tetBP*, *cmy−19, dfrF*, and *ant4−IIa* resistance gene types were the most abundant in the Y40, Y60, and Y80 groups. In the Y100 group, *aac6−Ie−aph2−Ia*, *sul3*, *dfrA12*, *qacH*, *cmlA6*, *aadA3*, *qnrS1*, *vgaC*, *ermF*, *tetW*, and *floR* were found to have the highest abundance. Lastly, *mdtO* and *pedo−1* resistance gene types have the highest abundance in the Y120 group. Other categories of drugs and resistance mechanisms were listed in Supplementary Table S1.

### 3.3. Representative Types of Antibiotics in Different Age Groups

**Figure 3:**
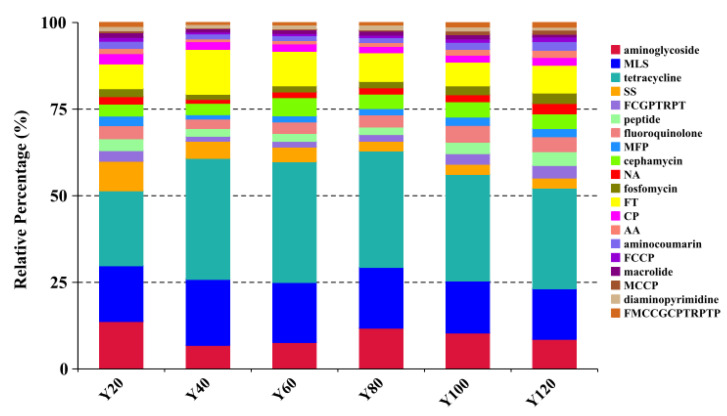
Comparison of the top 15 most abundant antibiotic types in the different age groups. The cumulative bar graph represents the distribution of the top 20 antibiotic resistance types in the different age groups.

We mapped each resistance gene type to its corresponding antibiotic, and calculated the sum of the relative abundances of antibiotic types (Figure 3). The results showed that tetracycline, MLS, aminoglycoside, FT, and sulfonamide and sulfone (SS) were the top five ARG types in each of the age groups, with tetracycline being the most abundant (Figure 3, Figure S3). At the same time, the trend of antibiotics consumption gradually increased with age. This was observed for antibiotics such as aminoglycoside and aminocoumarin (AA), fluoroquinolone, cephalosporin, glycylcycline, penam, tetracycline, rifamycin, phenicol, and triclosan (FCGPTRPT), fosfomycin, and peptide (Figure S3). Abbreviations of antibiotic types are listed in Supplementary Table S1.

### 3.4. Representative Antibiotic Resistance Types of Different Bacterial Genera

**Figure 4:**
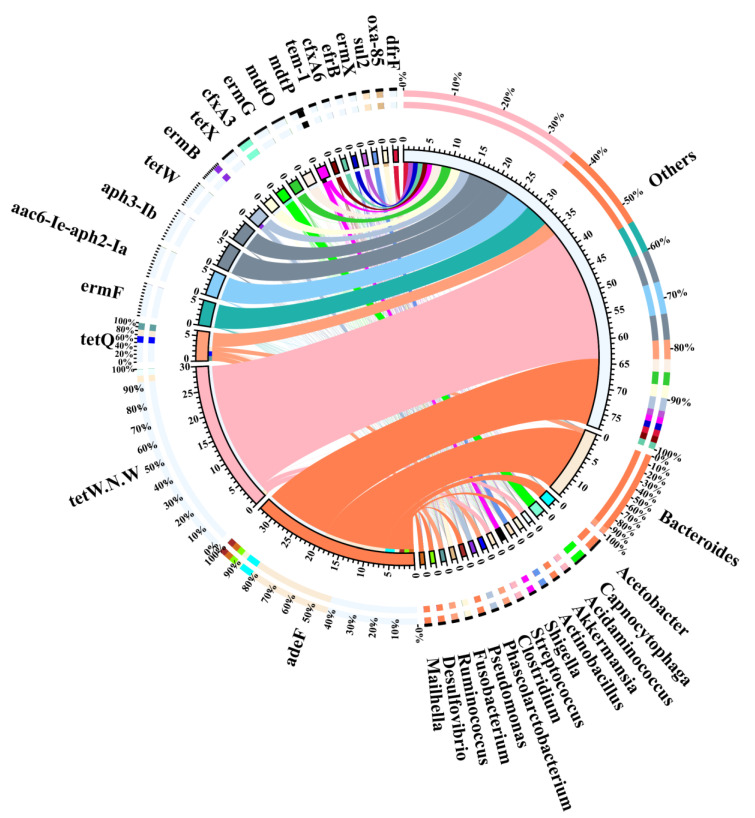
Analysis of the main bacterial genera carrying the top 20 antibiotic resistance gene types.

**Figure 5:**
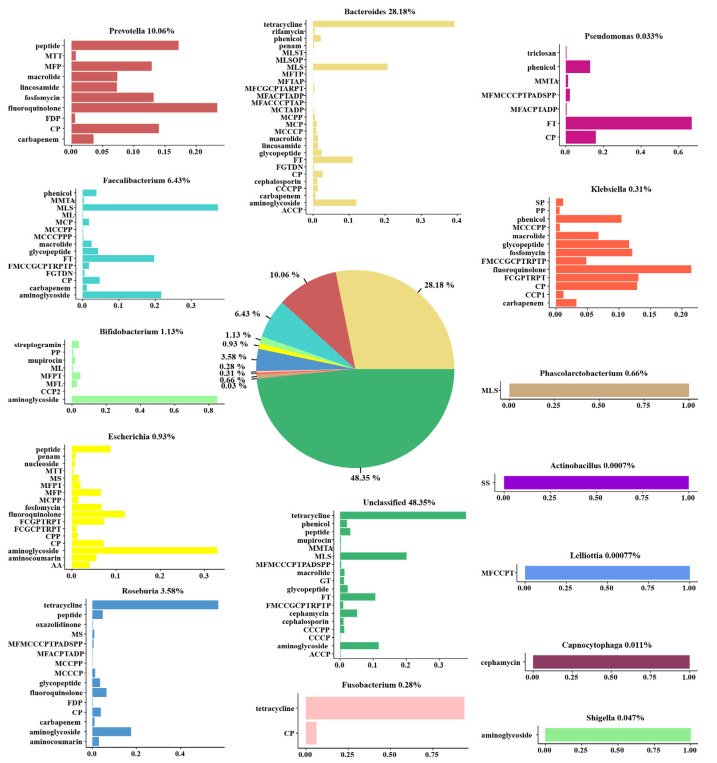
Major contributors to ARG. The pie chart represents the proportion of intestinal flora rich in ARG. The histograms represent the distribution of antibiotic resistance types in different bacterial genera.

To find out which bacterial genera contributed to the ARG reservoir, we performed an association analysis of ARG and bacterial genera. We found that the most predominant genus in the gut microbiota was *Bacteroides*, followed by *Firmicutes* and *Proteobacteria*. However, *Firmicutes* was the main carrier of antibiotic resistance gene bacteria, followed by *Proteobacteria* and *Bacteroides* (Figure S4). We observed that the distribution of bacteria rich in resistance genes at the phylum level was different between ARG and gut microbiota genes. This inconsistency indicates that, compared with other genes, ARG are less likely to appear in *Bacteroides*, but more likely to exist in *Firmicutes* and *Proteobacteria*. Interestingly, among the top twenty most abundant antibiotic resistance gene types, *Bacteroides* was the main carrier (Figure 4). The top ten contributing bacteria were *Bacteroides* (28.18%), *Prevotella* (10.06%), *Faecalibacterium* (6.43%), *Roseburia* (3.58%), *Bifidobacterium* (1.13%), *Escherichia* (0.93%), *Phascolarctobacterium* (0.66%), *Klebsiella* (0.31), *Fusobacterium* (0.28%), and *Pseudomonas* (0.03%). Similarly, the main carrier of ARG was *Bacteroides* (Figure 5).

We then characterised the antibiotic resistance types present in these bacteria. We observed that abundant antibiotic resistance types in *Bacteroides* were tetracycline resistance, followed by MLS. Similar to this, tetracycline was also the main antibiotic resistance type in *Roseburia*, *Fusobacterium,* and other unclassified bacteria (48.35%). Meanwhile, the main type of antibiotic resistance in *Prevotella* and *Klebsiella* was that of fluoroquinolone. The main type of antibiotic resistance in *Faecalibacterium* was MLS, followed by aminoglycoside. Remarkably, aminoglycoside was the main antibiotic resistance type in *Bifidobacterium*, *Escherichia,* and *Shigella* and was also widely distributed in *Bacteroides*, *Faecalibacterium,* and *Roseburia*. Lastly, we found that *Phascolarctobacterium*, *Actinobacillus*, *Lelliottia*, *Capnocytophaga,* and *Shigella* possessed only one type of antibiotic resistance (Figure 5).

## 4. Discussion

In this study, we characterised the reservoir of ARG in the human gut microbiota at the metagenomic level. We found that these genes were widespread in the microbiota and were more abundant and diverse in older (Y100 groups) individuals than in younger (Y20 and Y40 groups) ones. *Bacteroides* was revealed to be the main carrier of ARG, of which *tetQ* genes were the most abundant group. Antibiotic efflux, inactivation, and target alteration were the dominant mechanisms of resistance.

The detection of a large number of antibiotic resistance genes in the human gut microbiota has a technical reason that we cannot ignore. Due to the rapid development of high-throughput technology, which allows resistance genes that could not be sequenced on plasmids to be sequenced. The rapid change of bioinformatics analysis technology and the improvement of resistance gene database have made the resistance genes that were missed on the plasmids fully excavated. Therefore, the plasmid borne genes may have made a greater contribution.

The problem of microbial resistance to antibiotics can be attributed to several factors. It is an accepted fact that antibiotic abuse is the main reason for the development of resistance [55,56,57]. The difference in ARGs among the different age groups may be explained by the different selection pressures of antibiotics [58]. Based on previous studies, there is a direct correlation between antibiotic use and degree of resistance [59]. Antibiotic treatment disturbs the balance between the human host and its different microbes, leading to the emergence of antibiotic-resistant strains and related diseases [60,61]. In China, the abuse of antibiotics and the resulting problems of resistance are very serious. It is estimated that approximately 75% of seasonal flu patients take antibiotics [62]. In addition, compared with other countries, China has the fastest growth rate of resistance and the highest number and types of ARG [63,64]. It can be inferred that, due to the relatively weak supervision of antibiotics in China in the past few decades, antibiotics have been used in large quantities from childhood and accumulated over the life course. This may partly explain why the gut microbiota of the elderly in China have the largest number of ARG, although there are several other issues that need to be explored and traced, including how these resistance genes are human-related are acquired and spread.

Another contributing factor to the development of resistance are human-related activities, which may also explain how genes are acquired and spread. Antibiotics are widely used in activities such as livestock, agriculture, and aquaculture. Due to the increased demand for protein products, antibiotic use in livestock has increased significantly [65]. The persistent use of antibiotics in these contexts will increase the selective pressure for antibiotic resistance and the emergence of antibiotic-resistant strains, in which significant genetic exchange and recombination can occur and be easily transmitted to humans [66,67,68]. Bacteria from food, farm animals, and human clinical isolates can acquire antibiotic resistance through horizontal gene transfer [69], that is, antibiotic-resistant bacteria and ARG can be transmitted from animals to humans through various channels such as the food chain [70,71]. Forslund et al. demonstrated that the long-term use of antibiotics in livestock is a decisive factor for the high abundance of the ARG in the human gut microbiota [72].

The environment is also an important factor. As a variety of microorganisms are present in the environment, humans may interact with these microorganisms harbouring ARG directly or indirectly [73]. Antibiotics are not completely metabolized in the human body and may escape degradation and be excreted via urine and feces [74]. Since traditional wastewater treatment plants are not specifically designed to remove antibiotics, they are then discharged directly into the environment [75,76]. In addition, applying manure and sludge as fertilizer to soil, coupled with reclaimed water for irrigation, can promote the spread of antibiotics and ARG in the soil. Indeed, it has been shown that most of the ARG and genetic elements found in clinical isolates were also isolated in samples collected from wastewater [77,78]. Soil [79], sewage [80], and even air dust [81] may be important reservoirs involved in the spread of ARG. This suggests that the environment is a huge reservoir involved in the spread of ARG [82].

Finally, it should be noted that the problem of antibiotic resistance is not only due to external factors such as antibiotic abuse, but also internal mechanisms of bacteria that can cause antibiotics resistance—for example, enzymatic inhibition of antibiotic molecules, where bacteria can detoxify antibiotics by producing enzymes that can add specific chemical functional groups or destroy drugs through hydrolysis. Aminoglycoside-modifying enzymes can acetylate, phosphorylate, or adenylate aminoglycoside antibiotics so that they are unable to bind to bacterial ribosomal target sites [83]. Decreased antibiotic penetration can also occur [84]. For instance, a decrease in the number of porins can change the selectivity of the porin channel and limit drug uptake [85]. Efflux pumps are also an efficient antibiotic resistance mechanism as they actively remove antibiotics from the inside of the cell [86]. Lastly, target site modification can change the structure of the target such as the penicillin binding protein in the case of MRSA [87].

Together, these findings suggest that, in the human gut microbiota, the abundance of ARG is an age-related cumulative effect and different gene types are predominant in differently aged individuals. Several factors contribute to the development of ARG in the human gut microbiota; however, the extent to which these genes are affected by the factors needs to be further studied. Transformation of these factors may work synergistically with other factors, such as age, physical sex, and eating habits. Future research should seek to clarify the key role of hosts, carriers, and vectors in the transmission chain and determine the mechanisms that promote the spread of ARG between humans, the environment, and bacteria.

## Figures and Tables

**Table 1:** Top 20 antibiotic resistance genes in the gut microbiota.

ARO Name	Drug Class	Resistance Mechanism	AMR Gene Family
*tetQ*	tetracycline antibiotic	antibiotic target protection	tetracycline-resistant ribosomal protection protein
*adeF*	fluoroquinolone antibiotic; tetracycline antibiotic	antibiotic efflux	Resistance–nodulation–cell division RND antibiotic efflux pump
*ermF*	macrolide antibiotic; lincosamide antibiotic; streptogramin antibiotic	antibiotic target alteration	erm 23S ribosomal RNA methyltransferase
*ermB*	macrolide antibiotic; lincosamide antibiotic; streptogramin antibiotic	antibiotic target alteration	erm 23S ribosomal RNA methyltransferase
*tetW/N/W*	tetracycline antibiotic	antibiotic target protection	tetracycline-resistant ribosomal protection protein
*tetW*	tetracycline antibiotic	antibiotic target protection	tetracycline-resistant ribosomal protection protein
*sul2*	sulfonamide antibiotic; sulfone antibiotic	antibiotic target replacement	sulfonamide resistant sul
*cfxA3*	cephamycin	antibiotic inactivation	cfxA beta-lactamase
*aph3-Ib*	aminoglycoside antibiotic	antibiotic inactivation	aph3
*cfxA6*	cephamycin	antibiotic inactivation	cfxA beta-lactamase
*aac6-Ie-aph2-Ia*	aminoglycoside antibiotic	antibiotic inactivation	aph2; aac6
*tetX*	glycylcycline; tetracycline antibiotic	antibiotic inactivation	tetracycline inactivation enzyme
*dfrF*	diaminopyrimidine antibiotic	antibiotic target replacement	trimethoprim resistant dihydrofolate reductase dfr
*ermG*	macrolide antibiotic; lincosamide antibiotic; streptogramin antibiotic	antibiotic target alteration	erm 23S ribosomal RNA methyltransferase
*mdtO*	nucleoside antibiotic; acridine dye	antibiotic efflux	major facilitator superfamily MFS antibiotic efflux pump
*ermX*	macrolide antibiotic; lincosamide antibiotic; streptogramin antibiotic	antibiotic target alteration	erm 23S ribosomal RNA methyltransferase
*efrB*	macrolide antibiotic; fluoroquinolone antibiotic; rifamycin antibiotic	antibiotic efflux	ATP-binding cassette ABC antibiotic efflux pump
*mdtP*	nucleoside antibiotic; acridine dye	antibiotic efflux	major facilitator superfamily MFS antibiotic efflux pump
*oxa-85*	cephalosporin; penam	antibiotic inactivation	oxa beta-lactamase
*tem-1*	monobactam; cephalosporin; penam; penem	antibiotic inactivation	tem beta-lactamase

## Data Availability

The authors declare that all the data and the material used in this study are available within this article. All data generated or analysed during this study are available from the corresponding authors upon reasonable request.

## Supplementary Materials

The following are available online at https://www.mdpi.com/article/10.3390/antibiotics10081006/s1. Figure S1: The top twenty most abundant ARG types in the different age groups. Figure S2: Antimicrobial resistance mechanism of genes in gut microbiota. Figure S3: The box chart represents the distribution of a certain type of antibiotic resistance in the different age groups. Figure S4: Comparison of the distribution of the human gut ARG (inner cycle) and the microbiome gene set (outer cycle) at the bacterial phylum level. The ratios of genes (>1%) assigned to each phylum are shown in the pie charts. Table S1: Categories of drugs and resistance mechanisms.
