# Supplementary material for: Metagenomics-Based Analysis of the Age-Related Cumulative Effect of Antibiotic Resistance Genes in Gut Microbiota
Source: Antibiotics (Basel). 2021 Aug 20;10(8):1006. doi: 10.3390/antibiotics10081006 (PMC8388928; doi:10.3390/antibiotics10081006)
Supplement: Supplementary file 1 [file antibiotics-10-01006-s001.zip › Supplementary Table 1-2.pdf]

Supplementary Table S1 Categories of drugs and resistance mechanisms.

ARO name	Drug Class	Resistance Mechanism	AMR Gene Family
aph3-Ib	aminoglycoside antibiotic	antibiotic inactivation	aph3
sul2	sulfonamide antibiotic; sulfone antibiotic	antibiotic target replacement	sulfonamide resistant sul
ermB	macrolide antibiotic; lincosamide antibiotic; streptogramin antibiotic	antibiotic target alteration	erm 23S ribosomal RNA methyltransferase
tetQ	tetracycline antibiotic	antibiotic target protection	tetracycline-resistant ribosomal protection protein
ermG	macrolide antibiotic; lincosamide antibiotic; streptogramin antibiotic	antibiotic target alteration	erm 23S ribosomal RNA methyltransferase
ermF	macrolide antibiotic; lincosamide antibiotic; streptogramin antibiotic	antibiotic target alteration	erm 23S ribosomal RNA methyltransferase
cfxA3	cephamycin	antibiotic inactivation	cfxA beta-lactamase
adeF	fluoroquinolone antibiotic; tetracycline antibiotic	antibiotic efflux	resistance-nodulation-cell division RND antibiotic efflux pump
cfxA6	cephamycin	antibiotic inactivation	cfxA beta-lactamase
oxa-85	cephalosporin; penam	antibiotic inactivation	oxa beta-lactamase
ermX	macrolide antibiotic; lincosamide antibiotic; streptogramin antibiotic	antibiotic target alteration	erm 23S ribosomal RNA methyltransferase
tem-1	monobactam; cephalosporin; penam; penem	antibiotic inactivation	tem beta-lactamase
tetW	tetracycline antibiotic	antibiotic target protection	tetracycline-resistant ribosomal protection protein
aac6-Ie-aph2-Ia	aminoglycoside antibiotic	antibiotic inactivation	aph2; aac6
tetW/N/W	tetracycline antibiotic	antibiotic target protection	tetracycline-resistant ribosomal protection protein
efrB	macrolide antibiotic; fluoroquinolone antibiotic; rifamycin antibiotic	antibiotic efflux	ATP-binding cassette ABC antibiotic efflux pump
dfrF	diaminopyrimidine antibiotic	antibiotic target replacement	trimethoprim resistant dihydrofolate reductase dfr

mdtO	nucleoside antibiotic; acridine dye	antibiotic efflux	major facilitator superfamily MFS antibiotic efflux pump
tetX	glycylcycline; tetracycline antibiotic	antibiotic inactivation	tetracycline inactivation enzyme
mdtP	nucleoside antibiotic; acridine dye	antibiotic efflux	major facilitator superfamily MFS antibiotic efflux pump
escherichia coli soxR	fluoroquinolone antibiotic; cephalosporin; glycylcycline; penam; tetracycline antibiotic; rifamycin antibiotic; phenicol antibiotic; triclosan	antibiotic target alteration; antibiotic efflux	ATP-binding cassette ABC antibiotic efflux pump; major facilitator superfamily MFS antibiotic efflux pump; resistance-nodulation-cell division RND antibiotic efflux pump
tet40	tetracycline antibiotic	antibiotic efflux	major facilitator superfamily MFS antibiotic efflux pump
gadW	macrolide antibiotic; fluoroquinolone antibiotic; penam	antibiotic efflux	resistance-nodulation-cell division RND antibiotic efflux pump
emrD		antibiotic efflux	major facilitator superfamily MFS antibiotic efflux pump
escherichia coli ampC	cephalosporin; penam	antibiotic inactivation	ampC-type beta-lactamase
aadA5	aminoglycoside antibiotic	antibiotic inactivation	ant3
cpxA	aminoglycoside antibiotic; aminocoumarin antibiotic	antibiotic efflux	resistance-nodulation-cell division RND antibiotic efflux pump
escherichia coli soxS	fluoroquinolone antibiotic; monobactam; carbapenem; cephalosporin; glycylcycline; cephamycin; penam; tetracycline antibiotic; rifamycin antibiotic; phenicol antibiotic; triclosan; penem	antibiotic target alteration; antibiotic efflux; reduced permeability to antibiotic	ATP-binding cassette ABC antibiotic efflux pump; major facilitator superfamily MFS antibiotic efflux pump; resistance-nodulation-cell division RND antibiotic efflux pump; General Bacterial Porin with reduced permeability to beta-lactams
tetO	tetracycline antibiotic	antibiotic target protection	tetracycline-resistant ribosomal protection protein
aph6-Id	aminoglycoside antibiotic	antibiotic inactivation	aph6
acrB	fluoroquinolone antibiotic; cephalosporin; glycylcycline; penam; tetracycline antibiotic; rifamycin antibiotic; phenicol antibiotic; triclosan	antibiotic efflux	resistance-nodulation-cell division RND antibiotic efflux pump

mdtA	aminocoumarin antibiotic	antibiotic efflux	resistance-nodulation-cell division RND antibiotic efflux pump
escherichia coli CyaA	fosfomycin	antibiotic target alteration	cya adenylate cyclase
mdtN	nucleoside antibiotic; acridine dye	antibiotic efflux	major facilitator superfamily MFS antibiotic efflux pump
acrD	aminoglycoside antibiotic	antibiotic efflux	resistance-nodulation-cell division RND antibiotic efflux pump
cat	phenicol antibiotic	antibiotic inactivation	chloramphenicol acetyltransferase CAT
sul1	sulfonamide antibiotic; sulfone antibiotic	antibiotic target replacement	sulfonamide resistant sul
mdtE	macrolide antibiotic; fluoroquinolone antibiotic; penam	antibiotic efflux	resistance-nodulation-cell division RND antibiotic efflux pump
acrF	fluoroquinolone antibiotic; cephalosporin; cephamycin; penam	antibiotic efflux	resistance-nodulation-cell division RND antibiotic efflux pump
lnuC	lincosamide antibiotic	antibiotic inactivation	lincosamide nucleotidyltransferase LNU
aac3-IIa	aminoglycoside antibiotic	antibiotic inactivation	aac3
pmrC	peptide antibiotic	antibiotic target alteration	pmr phosphoethanolamine transferase
acrE	fluoroquinolone antibiotic; cephalosporin; cephamycin; penam	antibiotic efflux	resistance-nodulation-cell division RND antibiotic efflux pump
gadX	macrolide antibiotic; fluoroquinolone antibiotic; penam	antibiotic efflux	resistance-nodulation-cell division RND antibiotic efflux pump
bacA	peptide antibiotic	antibiotic target alteration	undecaprenyl pyrophosphate related proteins
dfrA17	diaminopyrimidine antibiotic	antibiotic target replacement	trimethoprim resistant dihydrofolate reductase dfr
aph3-IIIa	aminoglycoside antibiotic	antibiotic inactivation	aph3
haemophilus influenzae PBP3	monobactam; carbapenem; cephalosporin; cephamycin; penam	antibiotic target alteration	Penicillin-binding protein mutations conferring resistance to beta-lactam antibiotics

patA	fluoroquinolone antibiotic	antibiotic efflux	ATP-binding cassette ABC antibiotic efflux pump
mdtF	macrolide antibiotic; fluoroquinolone antibiotic; penam	antibiotic efflux	resistance-nodulation-cell division RND antibiotic efflux pump
emrR	fluoroquinolone antibiotic	antibiotic efflux	major facilitator superfamily MFS antibiotic efflux pump
acrS	fluoroquinolone antibiotic; cephalosporin; glycylcycline; cephamycin; penam; tetracycline antibiotic; rifamycin antibiotic; phenicol antibiotic; triclosan	antibiotic efflux	resistance-nodulation-cell division RND antibiotic efflux pump
escherichia coli acrA	fluoroquinolone antibiotic; cephalosporin; glycylcycline; penam; tetracycline antibiotic; rifamycin antibiotic; phenicol antibiotic; triclosan	antibiotic efflux	resistance-nodulation-cell division RND antibiotic efflux pump
mdtM	fluoroquinolone antibiotic; lincosamide antibiotic; nucleoside antibiotic; acridine dye; phenicol antibiotic	antibiotic efflux	major facilitator superfamily MFS antibiotic efflux pump
escherichia coli acrR	fluoroquinolone antibiotic; cephalosporin; glycylcycline; penam; tetracycline antibiotic; rifamycin antibiotic; phenicol antibiotic; triclosan	antibiotic target alteration; antibiotic efflux	resistance-nodulation-cell division RND antibiotic efflux pump
dfrA14	diaminopyrimidine antibiotic	antibiotic target replacement	trimethoprim resistant dihydrofolate reductase dfr
mphA	macrolide antibiotic	antibiotic inactivation	macrolide phosphotransferase MPH
imp-18	carbapenem; cephalosporin; cephamycin; penam; penem	antibiotic inactivation	imp beta-lactamase
tolC	macrolide antibiotic; fluoroquinolone antibiotic; cephalosporin; glycylcycline; cephamycin; penam; tetracycline antibiotic; aminocoumarin antibiotic; rifamycin antibiotic; phenicol antibiotic; triclosan	antibiotic efflux	ATP-binding cassette ABC antibiotic efflux pump; major facilitator superfamily MFS antibiotic efflux pump; resistance-nodulation-cell division RND antibiotic efflux pump
norB	fluoroquinolone antibiotic	antibiotic efflux	major facilitator superfamily MFS antibiotic efflux

			pump
escherichia coli UhpT	fosfomycin	antibiotic target alteration	uhpT
tetBP	tetracycline antibiotic	antibiotic target protection	tetracycline-resistant ribosomal protection protein
mrx	macrolide antibiotic	antibiotic inactivation	macrolide phosphotransferase MPH
emrA	fluoroquinolone antibiotic	antibiotic efflux	major facilitator superfamily MFS antibiotic efflux pump
emrB	fluoroquinolone antibiotic	antibiotic efflux	major facilitator superfamily MFS antibiotic efflux pump
kdpE	aminoglycoside antibiotic	antibiotic efflux	kdpDE
aph3-IIc	aminoglycoside antibiotic	antibiotic inactivation	aph3
evgA	macrolide antibiotic; fluoroquinolone antibiotic; penam; tetracycline antibiotic	antibiotic efflux	major facilitator superfamily MFS antibiotic efflux pump; resistance-nodulation-cell division RND antibiotic efflux pump
emrK	tetracycline antibiotic	antibiotic efflux	major facilitator superfamily MFS antibiotic efflux pump
mdtG	fosfomycin	antibiotic efflux	major facilitator superfamily MFS antibiotic efflux pump
tet41	tetracycline antibiotic	antibiotic efflux	major facilitator superfamily MFS antibiotic efflux pump
oxa-347	cephalosporin; penam	antibiotic inactivation	oxa beta-lactamase
qacH	fluoroquinolone antibiotic	antibiotic efflux	small multidrug resistance SMR antibiotic efflux pump
pmrF	peptide antibiotic	antibiotic target alteration	pmr phosphoethanolamine transferase
baeR	aminoglycoside antibiotic; aminocoumarin antibiotic	antibiotic efflux	resistance-nodulation-cell division RND antibiotic efflux pump
vgaC	streptogramin antibiotic; pleuromutilin antibiotic	antibiotic efflux	ATP-binding cassette ABC antibiotic efflux pump
mel	macrolide antibiotic; streptogramin antibiotic	antibiotic efflux	ATP-binding cassette ABC antibiotic efflux pump
arnA	peptide antibiotic	antibiotic target alteration	pmr phosphoethanolamine transferase
mdtC	aminocoumarin antibiotic	antibiotic efflux	resistance-nodulation-cell division RND antibiotic efflux pump
dfrA12	diaminopyrimidine antibiotic	antibiotic target replacement	trimethoprim resistant dihydrofolate reductase dfr

escherichia coli GlpT	fosfomycin	antibiotic target alteration	glpT
escherichia coli mdfA	tetracycline antibiotic; benzalkonium chloride; rhodamine	antibiotic efflux	major facilitator superfamily MFS antibiotic efflux pump
evgS	macrolide antibiotic; fluoroquinolone antibiotic; penam; tetracycline antibiotic	antibiotic efflux	major facilitator superfamily MFS antibiotic efflux pump; resistance-nodulation-cell division RND antibiotic efflux pump
cmy-19	cephamycin	antibiotic inactivation	cmy beta-lactamase
emrY	tetracycline antibiotic	antibiotic efflux	major facilitator superfamily MFS antibiotic efflux pump
h-NS	macrolide antibiotic; fluoroquinolone antibiotic; cephalosporin; cephamycin; penam; tetracycline antibiotic	antibiotic efflux	major facilitator superfamily MFS antibiotic efflux pump; resistance-nodulation-cell division RND antibiotic efflux pump
yojI	peptide antibiotic	antibiotic efflux	ATP-binding cassette ABC antibiotic efflux pump
floR	phenicol antibiotic	antibiotic efflux	major facilitator superfamily MFS antibiotic efflux pump
baeS	aminoglycoside antibiotic; aminocoumarin antibiotic	antibiotic efflux	resistance-nodulation-cell division RND antibiotic efflux pump
mdtH	fluoroquinolone antibiotic	antibiotic efflux	major facilitator superfamily MFS antibiotic efflux pump
aadA3	aminoglycoside antibiotic	antibiotic inactivation	ant3
mdtB	aminocoumarin antibiotic	antibiotic efflux	resistance-nodulation-cell division RND antibiotic efflux pump
ant4-IIa	aminoglycoside antibiotic	antibiotic inactivation	ant4
pedo-1	carbapenem	antibiotic inactivation	subclass B3 PEDO beta-lactamase
escherichia coli marR	fluoroquinolone antibiotic; cephalosporin; glycylcycline; penam; tetracycline antibiotic; rifamycin antibiotic; phenicol antibiotic; triclosan	antibiotic target alteration; antibiotic efflux	resistance-nodulation-cell division RND antibiotic efflux pump
cmlA6	phenicol antibiotic	antibiotic efflux	major facilitator superfamily MFS antibiotic efflux pump
sul3	sulfonamide antibiotic; sulfone antibiotic	antibiotic target replacement	sulfonamide resistant sul

escherichia coli gyrA	fluoroquinolone antibiotic; nybomycin	antibiotic target alteration	fluoroquinolone resistant gyrA
qnrS1	fluoroquinolone antibiotic	antibiotic target protection	quinolone resistance protein qnr
lra-3	cephalosporin; penam	antibiotic inactivation	subclass B3 LRA beta-lactamase

Supplementary Table S2 Abbreviations of antibiotic types.

**Abbreviations**	**Full name**
aminoglycoside	aminoglycoside
MLS	macrolide;lincosamide;streptogramin
tetracycline	tetracycline
SS	sulfonamide;sulfone
FCGPTRPT	fluoroquinolone;cephalosporin;glycylcycline;penam;tetracycline;rifamycin;phenicol;triclosan
peptide	peptide
fluoroquinolone	fluoroquinolone
MFP	macrolide;fluoroquinolone;penam
cephamycin	cephamycin
NA	nucleoside;acridine_dye
fosfomycin	fosfomycin
FT	fluoroquinolone;tetracycline
CP	cephalosporin;penam
AA	aminoglycoside;aminocoumarin
aminocoumarin	aminocoumarin
FCCP	fluoroquinolone;cephalosporin;cephamycin;penam
macrolide	macrolide
MCCP	monobactam;cephalosporin;penam;penem
diaminopyrimidine	diaminopyrimidine
FMCCGCPTRPTP	fluoroquinolone;monobactam;carbapenem;cephalosporin;glycylcycline;cephamycin;penam;tetracycline;rifamycin;phenicol;triclosan;penem
