# Supplementary figures and images for: Metagenomics-Based Analysis of the Age-Related Cumulative Effect of Antibiotic Resistance Genes in Gut Microbiota
Source: Antibiotics (Basel). 2021 Aug 20;10(8):1006. doi: 10.3390/antibiotics10081006 (PMC8388928; doi:10.3390/antibiotics10081006)

a

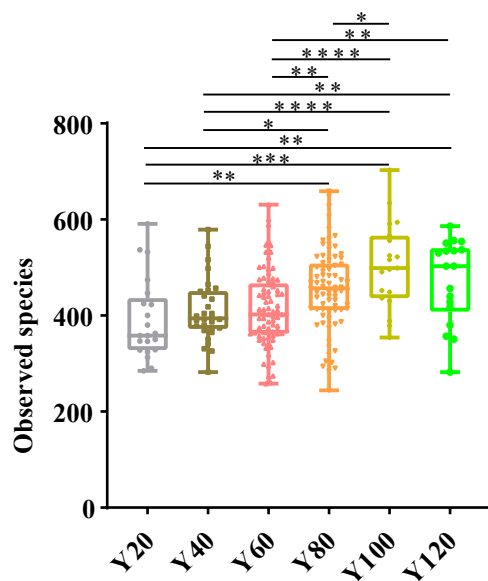

b

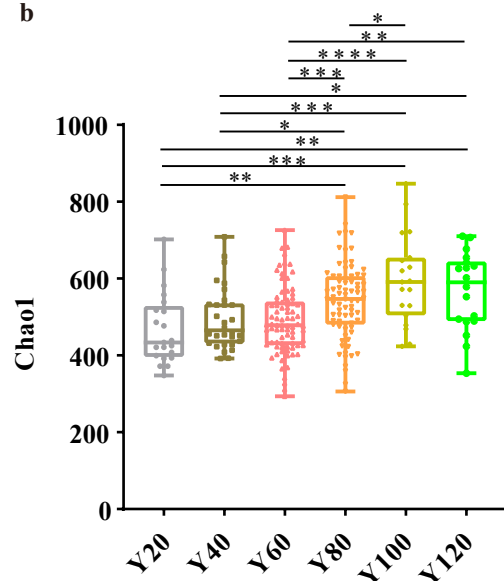

c

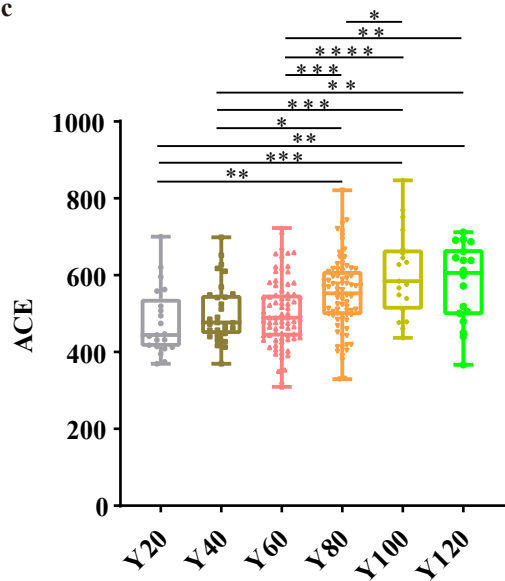

d

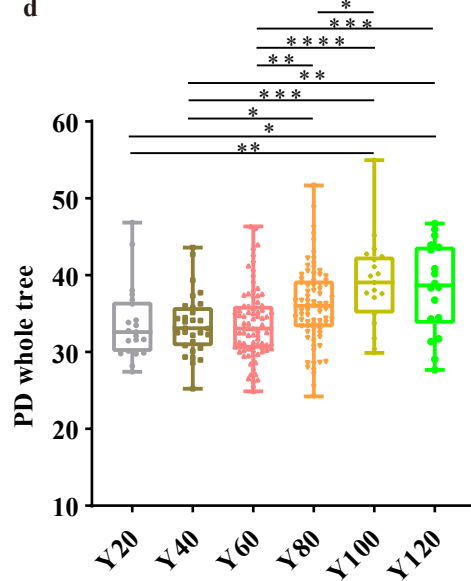

e

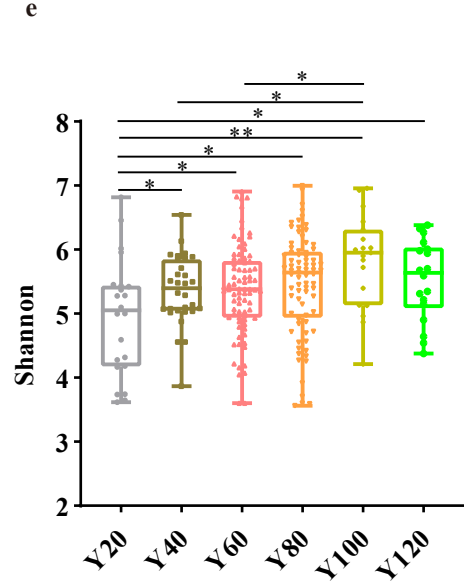

Supplement: Supplementary file 1 [file antibiotics-10-01006-s001.zip › Figure S1.pdf]

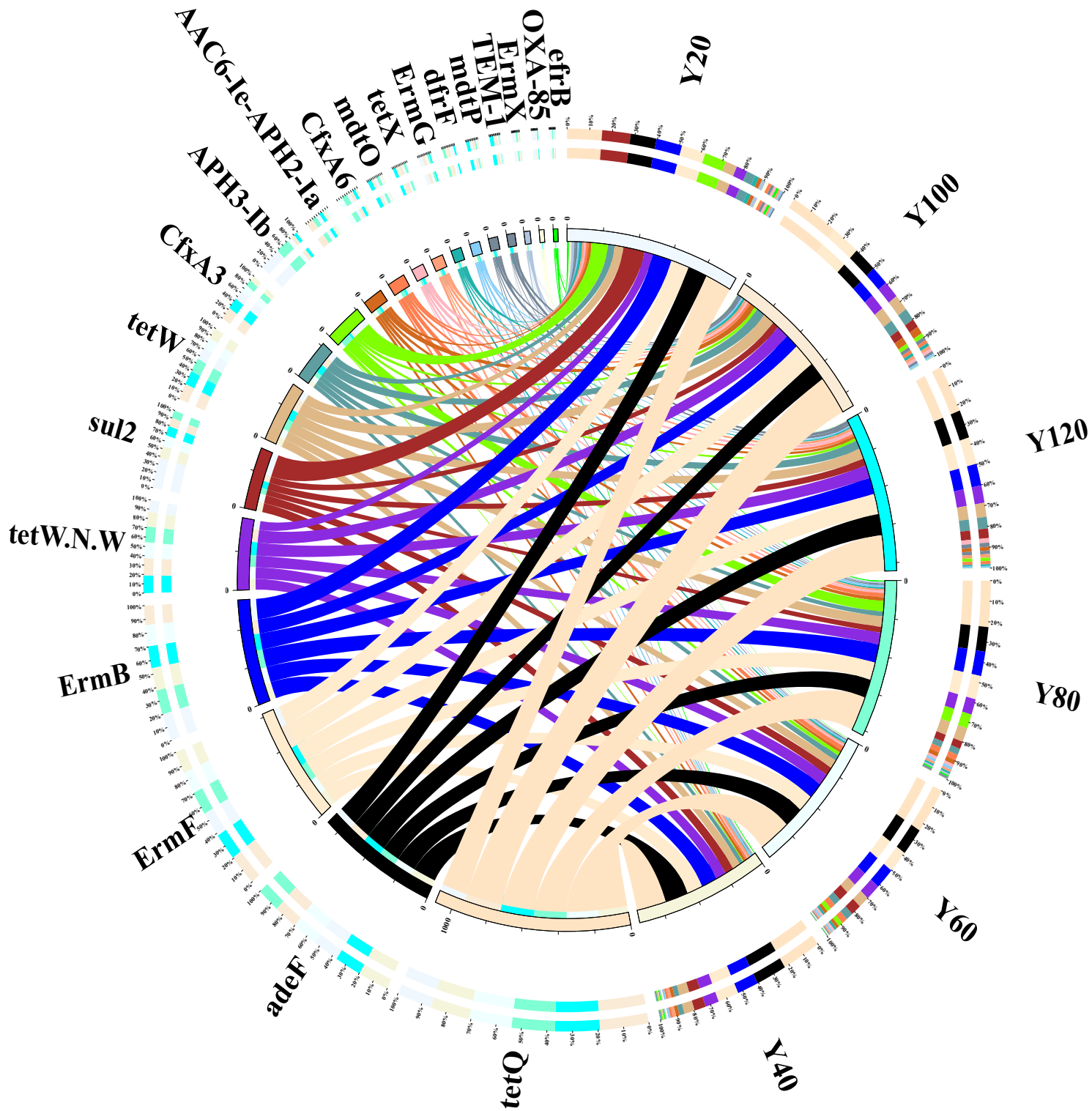

Supplement: Supplementary file 1 [file antibiotics-10-01006-s001.zip › Figure S2.pdf]

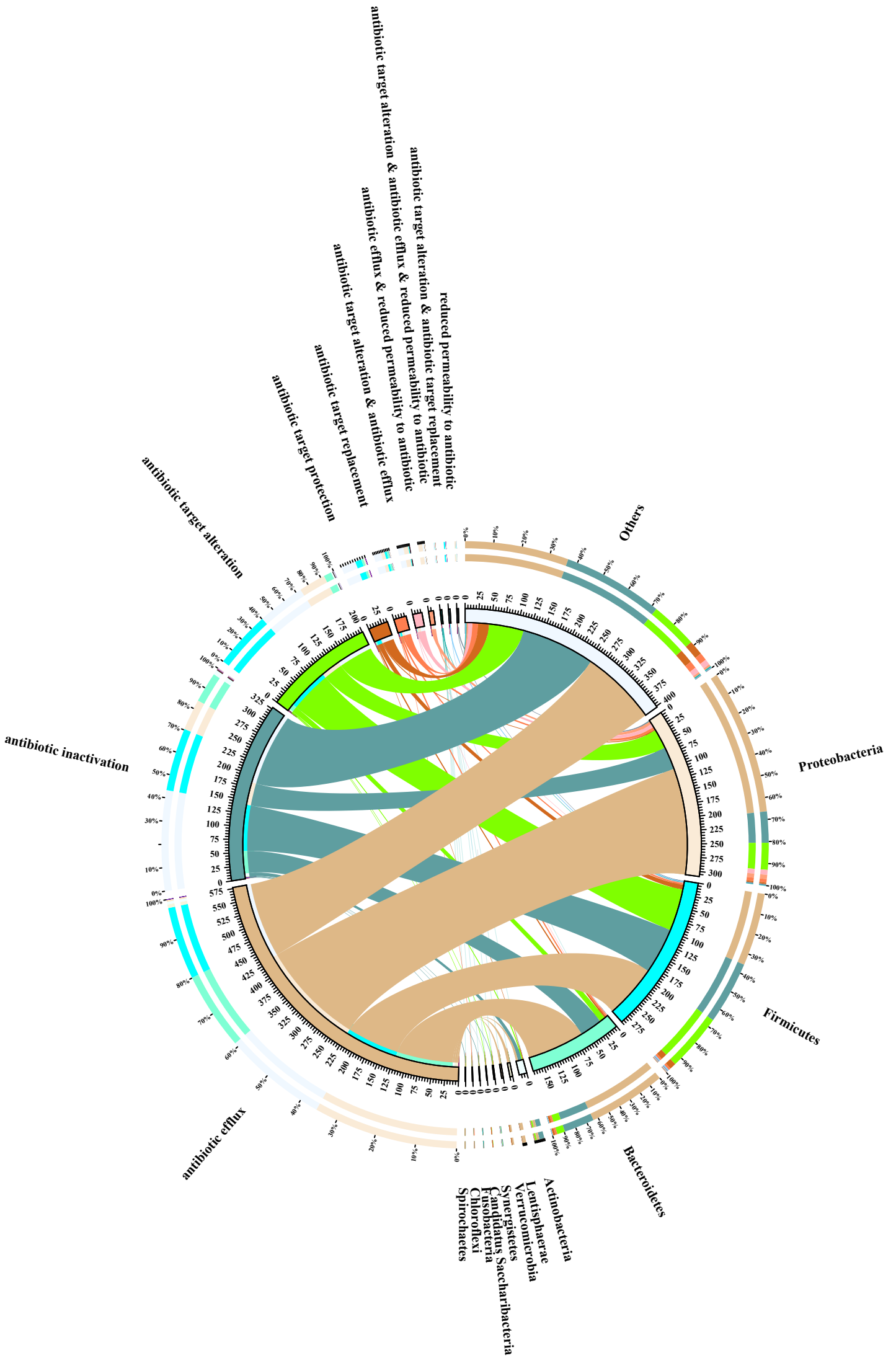

Supplement: Supplementary file 1 [file antibiotics-10-01006-s001.zip › Figure S3.pdf]

**a****Y20**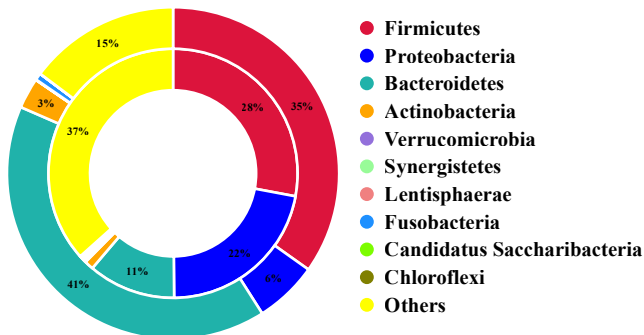**b****Y40**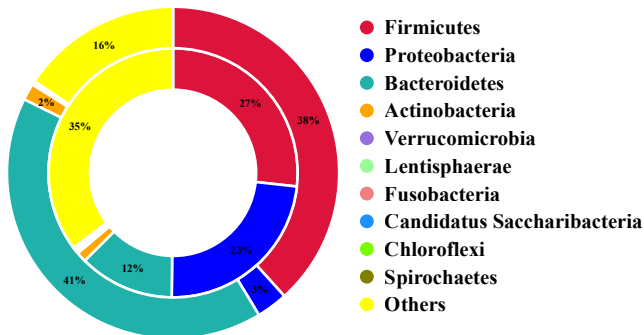**c****Y60**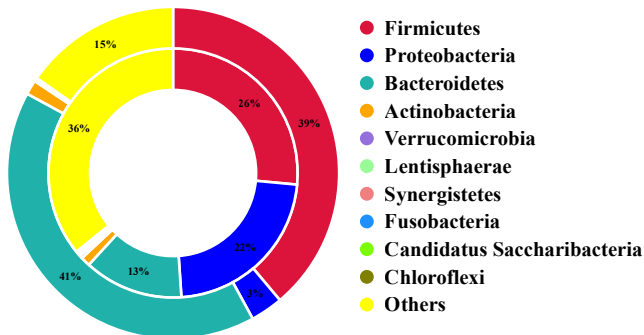**d****Y80**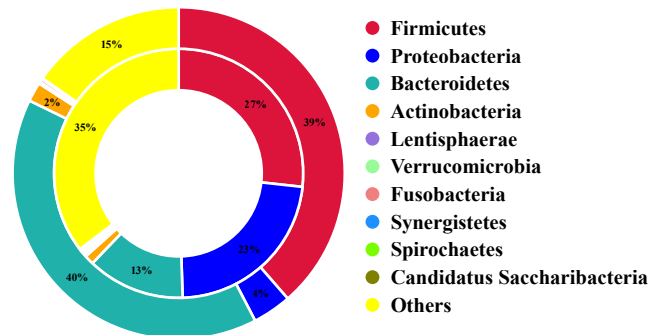**e****Y100**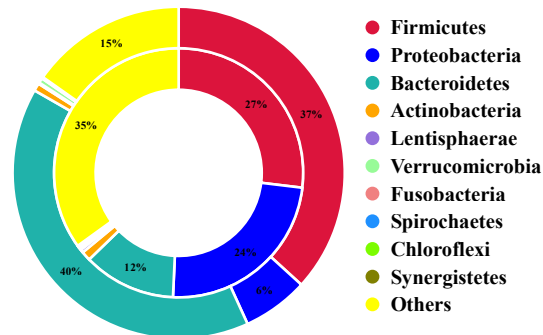**f****Y120**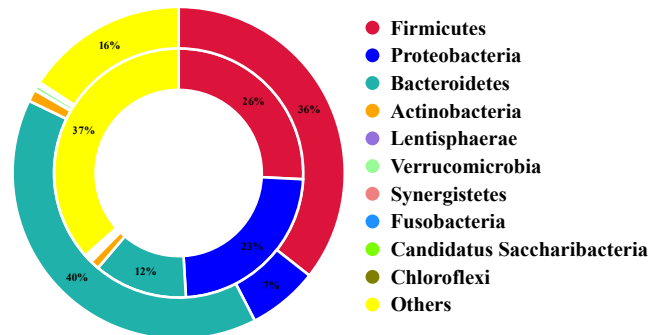

Supplement: Supplementary file 1 [file antibiotics-10-01006-s001.zip › Figure S4.pdf]
